# Normalization of High Dimensional Genomics Data Where the Distribution of the Altered Variables Is Skewed

**DOI:** 10.1371/journal.pone.0027942

**Published:** 2011-11-22

**Authors:** Mattias Landfors, Philge Philip, Patrik Rydén, Per Stenberg

**Affiliations:** 1 Computational Life Science Cluster (CLiC), Umeå University, Umeå, Sweden; 2 Division of Clinical Bacteriology, Umeå University, Umeå, Sweden; 3 Department of Mathematics and Mathematical Statistics, Umeå University, Umeå, Sweden; 4 Department of Molecular Biology, Umeå University, Umeå, Sweden; Inserm U869, France

## Abstract

Genome-wide analysis of gene expression or protein binding patterns using different array or sequencing based technologies is now routinely performed to compare different populations, such as treatment and reference groups. It is often necessary to normalize the data obtained to remove technical variation introduced in the course of conducting experimental work, but standard normalization techniques are not capable of eliminating technical bias in cases where the distribution of the truly altered variables is skewed, *i.e.* when a large fraction of the variables are either positively or negatively affected by the treatment. However, several experiments are likely to generate such skewed distributions, including ChIP-chip experiments for the study of chromatin, gene expression experiments for the study of apoptosis, and SNP-studies of copy number variation in normal and tumour tissues. A preliminary study using spike-in array data established that the capacity of an experiment to identify altered variables and generate unbiased estimates of the fold change decreases as the fraction of altered variables and the skewness increases. We propose the following work-flow for analyzing high-dimensional experiments with regions of altered variables: (1) Pre-process raw data using one of the standard normalization techniques. (2) Investigate if the distribution of the altered variables is skewed. (3) If the distribution is not believed to be skewed, no additional normalization is needed. Otherwise, re-normalize the data using a novel HMM-assisted normalization procedure. (4) Perform downstream analysis. Here, ChIP-chip data and simulated data were used to evaluate the performance of the work-flow. It was found that skewed distributions can be detected by using the novel DSE-test (Detection of Skewed Experiments). Furthermore, applying the HMM-assisted normalization to experiments where the distribution of the truly altered variables is skewed results in considerably higher sensitivity and lower bias than can be attained using standard and invariant normalization methods.

## Introduction

**Figure 1:**
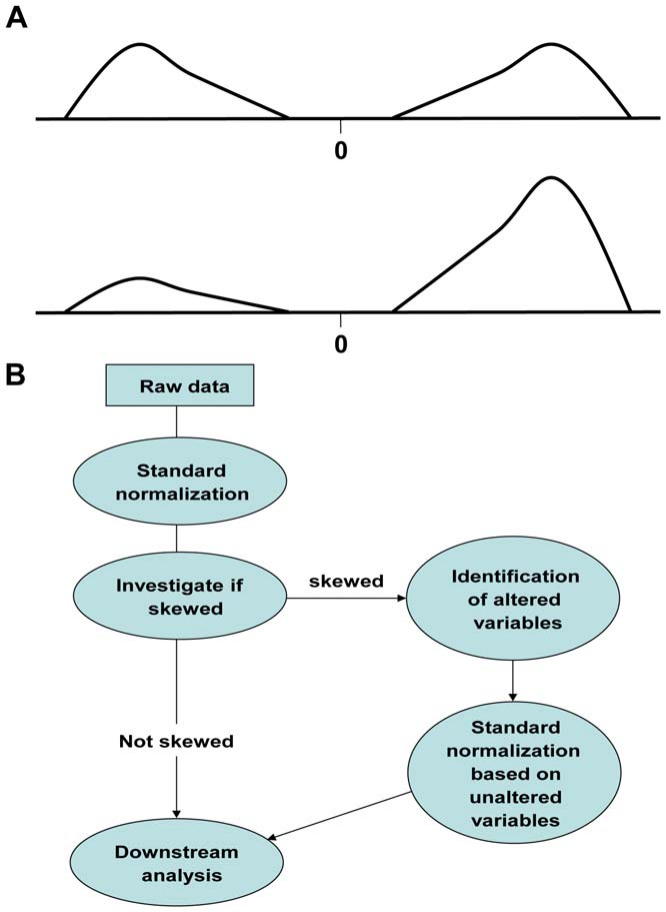
Skewed experiments and workflow. (A) The distribution of the *true log-ratios* of the *altered* variables in a non-skewed (upper) and a skewed (lower) experiment. Here an experiment with samples from a treatment and a reference population is considered and the true log-ratios are the expected value of the variables' log-ratios in the absence of any type of technical variation. (B) Our suggested workflow when analyzing data from high-dimensional experiments. Here the raw data is pre-processed and some kind of standard normalization is applied (*e.g.* quantile or MA-normalization). The normalized data is used to determine whether the experiment is skewed or not. For skewed experiments, a hidden Markov model is used to identify altered variables and then a standard normalization based on unaltered variables is used to normalize the data.

Genome-wide analysis of gene expression or protein binding patterns using different array or sequencing based technologies is now routinely performed in many molecular biology laboratories. Generally, biological replicates of treatment and control samples are compared in order to separate biologically relevant information from background variation. Before reference and treatment can be compared, some type of normalization needs to be applied because it is often the case that much of the observed variation reflects differences in the amount of material loaded or other technical variation. There are many well established procedures that can be used to normalize data. Typically, standard normalization methods, such as quantile normalization [1] and MA-normalization [2], will fail if; (1) a significant fraction of the variables are altered and (2) the distribution of the altered variables is not symmetrical, *i.e.* the distribution of the *true log-ratios* is not symmetrical around zero. The log-ratio is the logarithm of the ratio between the treatment and the control values. Here, the true log-ratios are the expected value of the log-ratios in the absence of any technical variation (Figure 1A shows the distribution of the true log-ratios in a symmetric and a skewed experiment). We say that an experiment is *skewed* if the distribution of the true log-ratios is not symmetrical around zero. For non-skewed experiments we expect an equal amount of *positively* and *negatively* affected variables. Here a *positively affected* variable is one for which the true log-ratio is positive. Using the terminology employed to describe ChIP-chip data and expression data, one would describe such a variable as being “enriched” or “up-regulated”.

For many experiments, the standard normalization methods (like quantile and MA-normalization) are perfectly suitable. However, in cases where the experiment is highly skewed, with a large fraction of altered variables, standard methods will most likely fail to remove the technical bias. As a result, the experiments' ability to identify altered variables and predict their fold change will be relatively low, leading to the loss of potentially relevant biological information [3]–[9]. We here use the term “skewed experiments” instead of “skewed data” to emphasise that we deal with data that is skewed because of the experimental setup and/or the nature of the biological problem, not because of technical bias and artefacts. It is hard to know how common such skewed experiments are; for a given experiment, it may be difficult to determine whether the assumption of symmetry is reasonable or not. However, it is clear that some routinely-performed high-dimensional experiments are likely to be skewed.

One experimental technique that is likely to generate highly skewed experiments is ChIP (Chromatin immuno-precipitation) followed by hybridization on tiling arrays (ChIP-chip) or followed by next generation sequencing (ChIP-seq). ChIP-chip experiments map a chromatin-bound protein or chromatin modification using specific antibodies and tiling arrays with probes that cover a large part of the genome. Antibodies specific for the protein or modification are added to a chromatin extract and bound chromatin is immuno-precipitated. The precipitated DNA is then extracted, amplified, labelled and hybridized to the tiling arrays. Typically, mock antibodies or input DNA are used as controls, which are hybridized to the same arrays. One would therefore expect numerous differences between the treated and control samples, especially if the protein or chromatin modification covers large parts of the genome. ChIP-chip and ChIP-seq experiments can therefore be assumed to be skewed. In many gene expression studies, only a small fraction of the genes are expected to be differentially expressed and consequently asymmetry is not an issue. However, it is likely to be relevant when studying cell death, heat-shock or hormonal treatment, all of which affect a relatively large fraction of the genome. If the fraction of up- and down-regulated genes differ, the experiment will be skewed. Tumour tissues and cell-lines commonly undergo extensive chromosomal rearrangements (see *e.g.* [10], [11]), and so skewness is to be expected when studying things such as the differences in copy number variation in tumorous and normal tissues. It should be noted that although the above experiments are likely to be skewed, they are commonly analyzed using standard normalization procedures; for instance, ChIP-chip data are often normalized using quantile normalization. Consequently, the normalization technique itself may introduce bias and thus result in the potential loss of relevant biological information. The skewness in these examples is a consequence of the biological phenomena being studied, and is not dependent on the technology used to acquire the data; it does not disappear if the array technologies are replaced by an alternative method such as a sequencing based technique. Normalization approaches that deal with skewed data have previously been proposed (see *e.g.* [3]–[9]), but to our knowledge there are no normalization methods that handle skewed experiments and that fully take advantage of the dependency structure (ordered variables which are dependent) that is present in many high-dimensional experiments.

We propose an approach that can be applied to any type of replicated high-dimensional experiment where two populations are compared. For all such experiments where normalization is necessary, we suggest the following work-flow: (1) Pre-process the raw data including some type of standard normalization. (2) Investigate if the experiment is skewed; by considering the experimental design, using visual inspection and applying the novel DSE-test (Detection of Skewed Experiments). (3a) If an experiment is not found to be skewed, no additional normalization is needed. (3b) If the experiment is found or believed to be skewed, the data is re-normalized using the novel HMM-assisted normalization. (4) Perform downstream analysis, *e.g.* identification of altered variables, classification or cluster analysis (the approach is summarized in Figure 1B).

Spike-in array data were used to study how skewness affects the capacity of an experiment to identify altered variables (*i.e.* its sensitivity) and generate unbiased estimates of the fold change of individual variables. It is shown that asymmetry can have a considerable negative effect on both bias and sensitivity. Simulated data and three tiling-array data sets were used to study the performance of our suggested work-flow; including the DSE-test and the HMM-assisted normalization. The results suggest that the DSE-test combined with visual inspection can be a powerful approach to detect skewed experiments, even when applied to relatively small experiments. The HMM-assisted normalization uses a Hidden Markov Model (HMM) to identify regions that are unaltered. Variables identified as unaltered are used to estimate the normalization function and to normalize the entire data set without introducing any bias. The performance of the HMM-assisted normalization exceeded that of commonly used standard and invariant approaches in terms of sensitivity and bias. For some experiments, the HMM-assisted normalization had more than twice as high sensitivity as the standard quantile normalization. Although we focus on one-colour tiling array data, our work-flow can be applied to any data set with dependent variables, irrespective of the platform or protocol with which it was generated.

## Results and Discussion

Standard normalizations are often able to remove technical variation when two *primary assumptions* are valid, *i.e.* that only a small fraction of the variables are affected by the treatment and that the true log-ratios are approximately symmetrically distributed (see top graph in Figure 1A). If both assumptions are violated most commonly used normalization algorithms will fail. In principal, standard normalization methods involve two separate steps: first, the sample data *x* is used to estimate a *normalization function f*; second, the normalized data 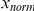 is obtained as 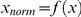. Importantly, the last step is not sensitive to the primary assumptions. An *ideal normalization* would only use data from non-altered variables when deriving the function *f* and obtain the normalized data as 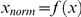. Several *invariant* normalization methods, aiming to identify a set of non-altered variables in order to obtain an unbiased estimate of the normalization function *f*, have been suggested; see *e.g.* [9]. There are also some alternative methods that cannot be regarded as invariant, but still addresses the problem of normalizing skewed experiments, *e.g.* the modified loess [5]. However, to our knowledge none of them take advantage of the dependency structure that is commonly present in *e.g.* ChIP-chip and RNA-sequencing experiments. Here, we suggest an HMM-based approach that can be applied on data with a dependency structure. The approach is an invariant method that uses a hidden Markov model to identify a set of non-altered variables and that can be used in conjunction with almost any of the standard normalization techniques.

### Violating the primary normalization assumptions affects bias and sensitivity

Spike-in data from an array experiment designed and conducted in-house [12] using 16 samples were used to evaluate how violations of the primary assumptions affect the experiment's *bias* (*i.e.* the ability to provide accurate estimates of fold change) and its *sensitivity* (*i.e.* the ability to identify affected variables) obtained at a reasonable false positive rate (0.5%). The arrays contained ∼7760 clones, of which 1920 were spiked so as to be up- or down-regulated by a factor of three relative to the control; see Materials and Methods for further details.

Five types of normalization procedures were evaluated: a) The standard one-channel quantile normalization [1]. b) The cyclic MA-loess normalization [1]. Both these methods are *standard* normalizations where all genes affect the estimate of the normalization function. c-d) The rank invariant normalization as suggested by Pelz, et al. [9], combined with either one-channel quantile or cyclic MA-loess normalization. e) The modified loess normalization suggested by Risso et al. [5]. Henceforth, we will refer to these methods as standard quantile, standard MA, invariant quantile, invariant MA and modified loess normalization. All methods were compared to *ideal quantile normalization* where only non-regulated clones were used when estimating the normalization function. The quantile normalization is frequently used for normalizing one-channel data, *e.g.* microarray and ChIP-chip data. An alternative to the ideal quantile normalization is the ideal cyclic MA loess normalization. The reason for choosing the ideal quantile normalization rather than the ideal cyclic MA loess normalization as a reference is that the former had considerably higher sensitivity; see Figure S1.

All five normalization procedures were applied on the spike-in data and all clones were normalized. Here, the percentage of altered clones was varied between 5 and 20% and the percentage of up-regulated clones among the altered clones was varied between 50 and 100%. The procedures were evaluated by considering their *relative sensitivity* and their *relative bias*, *i.e.* the procedures' performances relative the ideal quantile normalization; see Materials and Methods for further details. We believe that sensitivity and bias are important measures for evaluating the performance of different normalizations. Sensitivity is important when *e.g.* identifying protein targets or differentially expressed genes and bias may influence further downstream analyses. For example, regulatory networks inferred from expressional correlations, cluster analysis and classification may be affected by bias [13].

**Figure 2:**
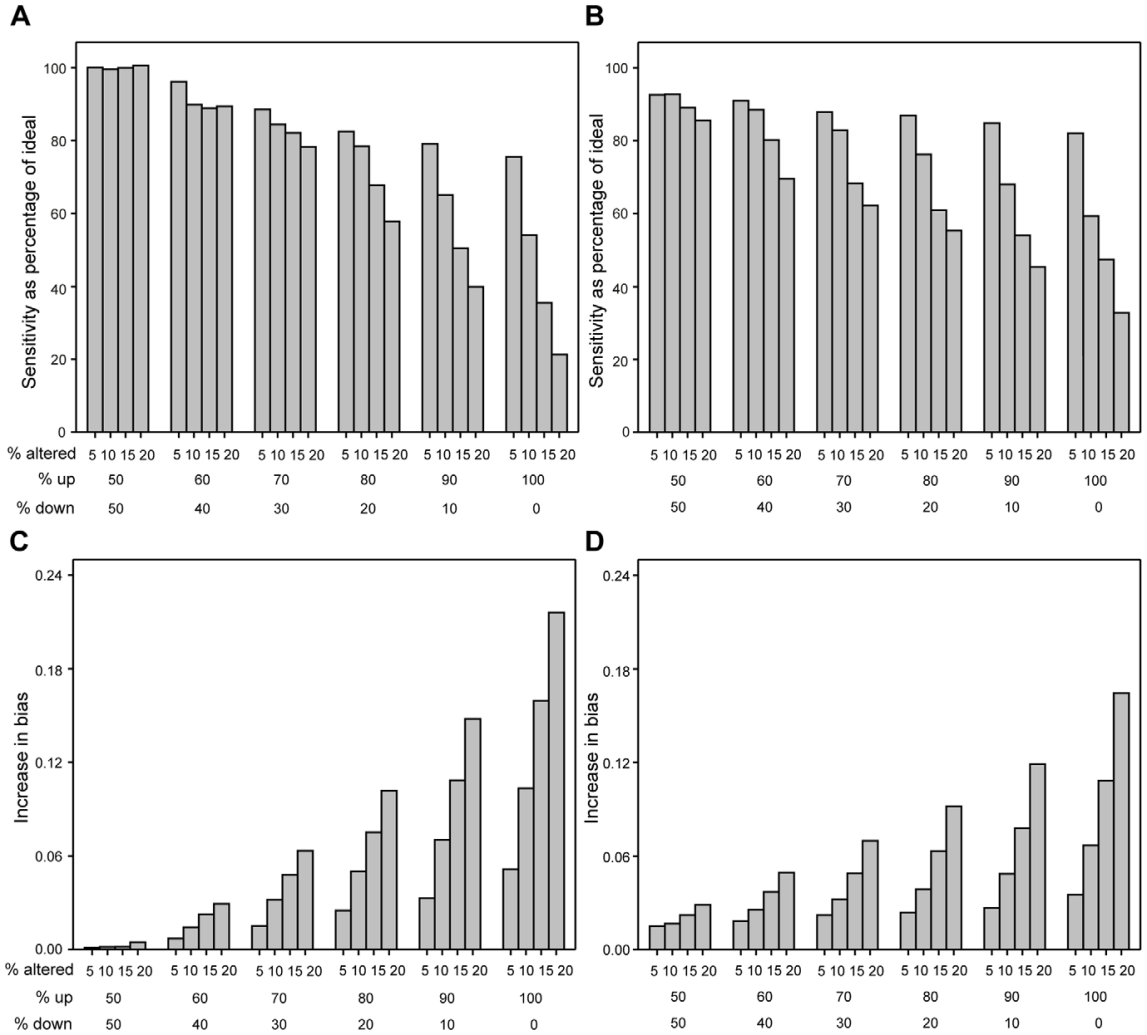
The effect of violating the primary assumptions. The sensitivity and bias for the standard quantile normalization and the rank invariant quantile normalization compared to the *ideal quantile normalization* (*i.e.* a quantile normalization where only the non-regulated clones influenced the normalization) for different percentages of altered clones (% altered) and different distributions of up- and down-regulated clones. (A) The relative sensitivity of the standard quantile normalization observed at 0.5% false positive rate (*i.e.* the ratio between the sensitivity observed when the standard and ideal quantile normalization was applied to the data). (B) The relative sensitivity of the invariant quantile normalization at 0.5% false positive rate. (C) The difference in bias between the standard and ideal quantile normalization (D). The difference in bias between the invariant and ideal quantile normalization.

The fraction of altered clones and the fraction of up-regulated clones among the altered clones (*i.e.* the experiment's skewness) have negative and synergistic effects on both bias and sensitivity (Figure 2 and Figure S2). This holds true for both the standard and invariant normalizations, although the invariant methods performs better than the standard methods when experiments are heavily skewed (*i.e.* 80–100% of the altered clones are up-regulated).

Interestingly, the standard methods performed better than the invariant methods when the experiments were symmetric (*i.e.* 50% of the altered clones are up-regulated) (Figure 2 and Figure S2). The result suggests that invariant methods should not be used in cases where the regulated clones are symmetrically distributed around zero.

The modified loess procedure failed to normalize the spike-in data and the observed sensitivities were very low (<10%) for all the considered parameter settings, despite the fact that the modified loess was the only method making use of the fact that the spike-in data was generated using two-channel arrays. The modified loess assumes that there is a limited amount of systematic technical variation (*e.g.* dye-bias) in the data [5]. This assumption is not valid for the spike-in data and may explain the failure. Moreover, we argue that for small one-channel experiments (*e.g.* two-four biological replicates) there is a considerable risk that we will have substantial systematic variation between the treatments.

We believe that the impact of experiments' skewness has largely been overlooked and, on the basis of our evaluation of the spike-in data, that the biological interpretation of experimental data could potentially be facilitated by adopting alternative normalization procedures.

### Investigating if an experiment is skewed

As discussed in the Introduction section several high-dimensional experiments are likely to be skewed. Often it is relatively easy to conclude that an experiment is skewed given the design and the experimental setup. In such cases no further investigation is necessary and the data is normalized using a technique that handles skewed experiments, *e.g.* the HMM-assisted normalization discussed in the next section. For other experiments it may be difficult to determine whether the assumption of symmetry is reasonable or not. For these experiments we suggest a two stage approach for detecting skewed experiments including visual inspection of the data and a novel test, called the *DSE-test* (Detection of Skewed Experiments).

### Visual Inspection

Initially, all samples are normalized using a standard normalization technique of the user's choosing. The averages of the variables' normalized values are calculated for the treatment and the reference groups separately. The variables' *M-values* (*i.e.* the log-ratio of the groups' averages) are then calculated for all variables. A visual inspection of the M-values estimated density function can be used to investigate if the experiment is skewed or not. For a non-skewed experiment we expect the distribution of the M-values to be fairly symmetrical.

It should be noted that it may be difficult to see if a distribution is skewed or not. In the case the visual inspection does not give any clear indication of skewness the investigation will continue by applying the DSE-test.

### The DSE-test

We propose a novel test to detect skewed experiments. Briefly, the idea behind the test is the following. Initially, all samples are normalized using a normalization technique of the user's choosing. For any pair of samples the variables' log-ratios can be calculated and the skewness of their distribution can be estimated using the quartile skewness coefficient (*qs-coefficient*) [14]. The test compares qs-coefficients obtained from *heterogeneous pairs* (one treated and one reference sample) against *homogeneou*s *pairs* (either two treated samples or two reference samples). If the heterogeneous qs-coefficients deviate significantly from the homogeneous qs-coefficients, then the experiment is said to be skewed; see Methods for further details.

Here, we propose two variants of the DSE-test; the independent variant (for which the constructed pairs are independent) and the dependent variant (for which some of the pairs are dependent). The independent test controls, in contrast to the dependent test, the false positive rate, but has for small experiments considerably lower power since fewer pairs can be constructed; see Methods for further details. We argue that for small experiments (*i.e.* less than 8 control and 8 reference samples) the power gain using the dependent DSE-test instead of the independent DSE-test may out-weight the potentially increased risk of having false positives.

To make our tests readily available we have created a user friendly web-application that can be used to test for skewness in any type of experiment including expression data, SNP-data and ChIP-chip data; see Materials and Methods for further details.

The performances of the independent and dependent DSE-tests were evaluated in a simulation study were data from skewed experiments with no negatively-affected variables were simulated. The aim was to generate simulated data that closely resembled real normalized data, but simulating realistic data is extremely difficult. In our simulations, we assumed that the variables' intensities were independent and normally distributed. Furthermore, we assumed that the *effect sizes* (*i.e.* the true ratios) of the altered variables were all the same. None of these assumptions are realistic for high-dimensional data sets that it would be most desirable to apply this test to, and so it must be stressed that the test's *power* (*i.e.* its probability of detecting skewed experiments) when applied to real data may be lower than was observed in this simulation study.

We simulated data for a wide range of experiments. The percentage of positively affected variables varied between 0–25%, the number of variables ranged between 10,000 and 100,000, the number of biological replicates per treatment ranged from 2 to 16, and the effect size was between 1.3 and 4. In total, 240 parameter combinations were evaluated and the test's power was estimated for each combination; see Methods for further details.

The power of the tests increased with the number of samples, variables, effect size, and the fraction of affected variables. For small experiments the dependent DSE-test has considerably higher power than the independent DSE-test (Tables S1 and S2). Interestingly, the power of the dependent DSE-test was at least 90% when the fraction of affected variables was at least 10%, the experiments had 100,000 variables, and the effect size was at least 2. This suggests that that the test may even be useful for very small high dimensional experiments (with as few as two control and two treatment samples), *e.g.* small experiments using tiling arrays or next generation sequencing data, in which the number of variables is normally very large (>1,000,000).

Visual inspection and the dependent DSE-test was applied to three real ChIP data sets, two of which were originally reported by Schwartz et al. [15] (E(z) and H3K27me3) and one by Nègre et al. [16] (PolII). All three of these data sets feature two control biological replicates generated using input DNA and two treatment replicates generated by ChIP; all samples were hybridized to Affymetrix Drosophila tiling arrays. The E(z) data set is a ChIP-chip data set with localized peaks; the histone modification H3K27me3 and PolII data sets span broad regions of the genome. Here, all three data sets were generated using the ChIP-chip technique, and we expect all of them to be skewed.

**Figure 3:**
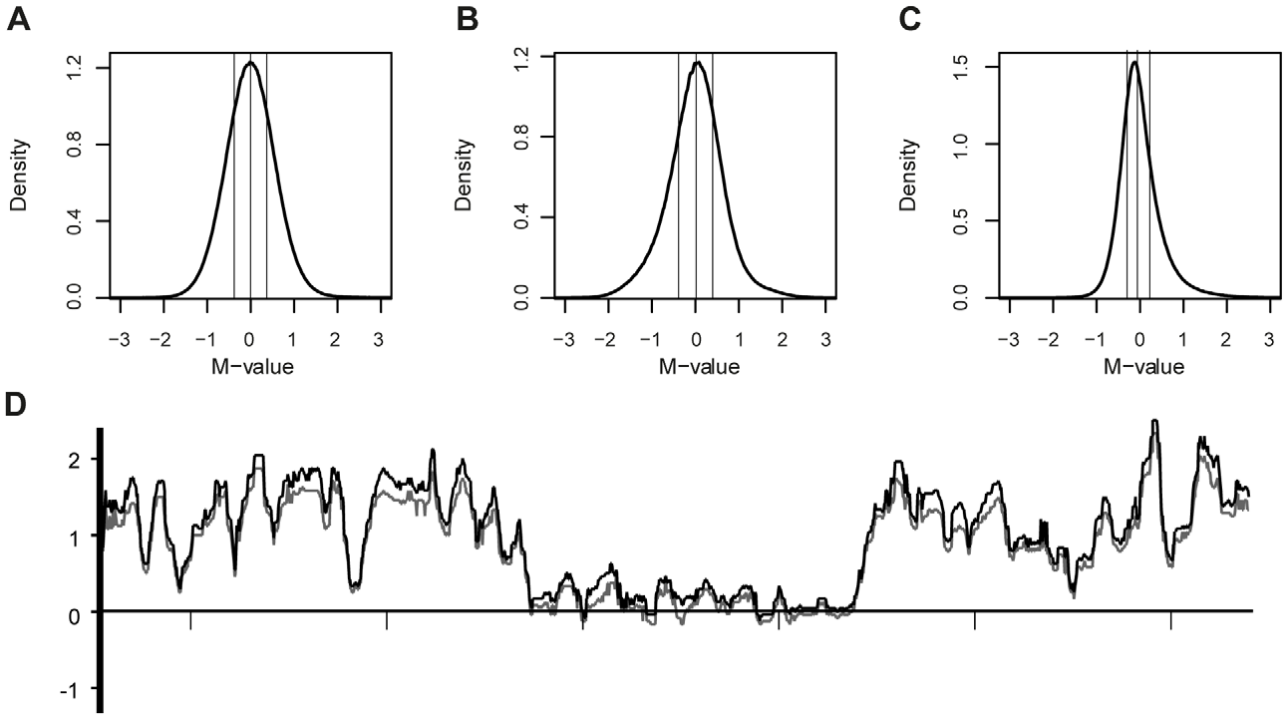
Visual inspection and normalization of ChIP-chip experiments. Visual inspection of three ChIP-chip experiments; (A) E(z), (B) H3K27me3 and (C) PolII. The estimated density functions (using the R-function *density* with the default bandwidth) of the observed M-values obtained after standard quantile normalization are shown. Vertical lines indicate first quartile, median and third quartile. (D) HMM-assisted normalization of H3K27me3 data. M-values after standard quantile normalization (grey) and HMM-assisted normalization (black). 10 Kb intervals are marked on the x-axis.

Visual inspections of the distributions of the M-values suggest that the PolII-experiment and arguably the H3K27me3-experiment are skewed, but that the E(z)-experiment is not particularly skewed (see Figure 3A–C). The PolII data set was found to be skewed according to the dependent DSE-test (p = 0.034), but the skewness of the H3K27me3 (p = 0.289) and E(z) (p = 0.997) experiments was found to be not-significant at the 5%-level. Again, we stress that the experiments are based on only two replicates and that the absence of a low p-value does not imply the experiments are not skewed. In general, we recommend the HMM-assisted normalization to be used on all experiments that for some reason are suspected to be skewed.

### HMM-assisted normalization of skewed data

We consider highly skewed experiments where a majority of the altered variables are either positively or negatively affected and where the fold changes of nearby variables are positively correlated. Our suggested normalization approach (from here referred to as HMM-normalization) is as follows: Initially, all samples are normalized using some type of standard normalization, *e.g.* quantile normalization. The averages of the variables' normalized values are calculated for the treatment and the reference groups separately. The variables' *M-values* (*i.e.* the log-ratio of the groups' averages) are then calculated for all variables. A hidden Markov model with two states is applied to the M-values and the variables belonging to the state whose mean is closest to zero are classified as unaltered variables. The samples are then re-normalized as in the first step, with the difference that only variables identified as being unaltered are allowed to influence the normalization function (but all variables are normalized); see Materials and Methods for further details. The application of hidden Markov models to ChIP data to identify *enriched regions* has previously been suggested [17]–[19]. We stress that our approach focuses on normalization and can be combined with any type of test for identification of altered variables.

The performance of the HMM-assisted normalization was evaluated in a simulation study, using part of the simulated data that was generated to evaluate the DSE-test. In total, data from 120 (we show the results where region length was set to 50, *m* = 50, since the results was virtually identical for *m* = 100) different experiments were simulated (see Materials and Methods for further details). The HMM-normalization was compared to a *standard normalization* (*i.e.* standard quantile normalization) and an *invariant normalization* (*i.e.* invariant quantile normalization). The methods were evaluated in terms of their utility in downstream analysis, in which the objective was to identify altered variables; the performance of the two methods was characterised in terms of the experiments' sensitivity, specificity, and bias. For each experiment and normalization, these characteristics were estimated for a fixed cut-off; see Materials and Methods for further details. The results generated using the evaluated approaches were compared to those obtained using the *ideal normalization* (*i.e.* ideal quantile normalization). Clearly, the ideal normalization cannot be used on real data, but it serves as a useful positive control.

**Figure 4:**
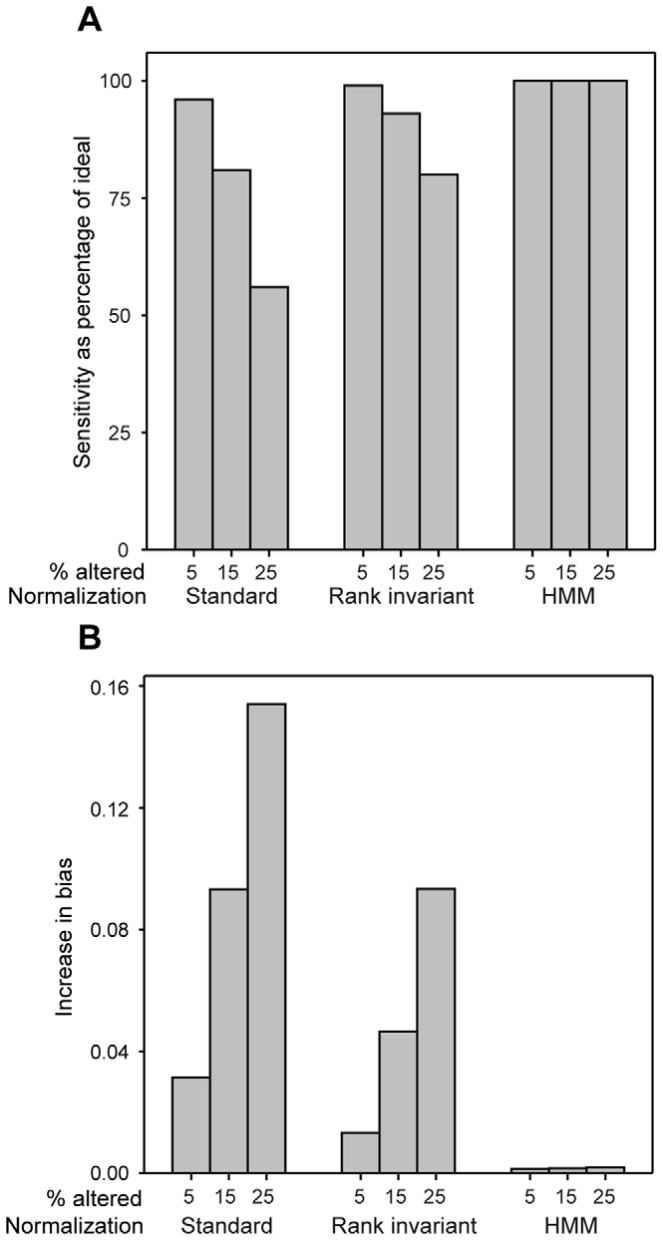
The performance of the HMM-assisted normalization. Comparison of the performance (sensitivity and bias) of the HMM, rank invariant and standard quantile normalizations relative the ideal quantile normalization evaluated on data simulated from skewed experiments. Three different percentages of altered variables (% altered) were considered (5, 15 and 25%). The experiments contained data from two treated samples and two reference samples, each with 100,000 variables. The altered variables were positively affected, with an effect size *δ* = 1.5 and was distributed in homogeneous regions including 50 altered probes (*i.e. m* = 50). A variable with an average M-value above the cut-off was called altered. For each experiment, the estimated sensitivity and bias was based on 10 simulated data sets. (A) The relative sensitivity for the HMM, invariant and standard quantile normalizations. Here, the relative specificity for the HMM, invariant and standard quantile normalizations was close to 100% for all considered parameter settings. (B) The difference in bias between the HMM, invariant and standard quantile normalization compared to the ideal quantile normalization.

Both the HMM and the invariant normalization performed considerably better than the standard normalization, with the former methods having higher sensitivity (at a similar or higher specificity) and lower bias than the standard normalization (Figure 4 and Tables S3, S4, S5). The relative gain achieved using the HMM-normalization compared to the standard normalization was rather extreme in some cases. For example, when 25% of the variables were positively altered, the relative sensitivity attained using HMM-normalization was 1.5–8 times higher than that achieved with the standard approach while the corresponding specificities were similar (Tables S4 and S5).

The overall performance of the HMM-normalization was close to that of the ideal normalization for all considered parameter settings (Figure 4 and Tables S3, S4, S5). Although the invariant normalization performed rather well, in particular for medium and large size experiments (more than 4 replicates per treatment) with a high effect size (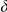
>1.5), it did not perform as well as the HMM-normalization. For all considered parameter settings the HMM-normalization did have higher (or equal) sensitivity and lower bias compared to the invariant normalization, while the corresponding specificities were similar. The differences between the two approaches increases as: the percentage of altered variables increases, the effect size decreases and the size of the experiment decreases (Figure 4 and Tables S3, S4, S5). For small experiments (not more than 4 replicates per treatment) with a low effect size (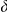 = 1.3 and 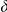 = 1.5) and 25% altered variables, the HMM-normalization had 1.1–1.4 times larger sensitivity than the invariant normalization, while the observed specificities were similar. For these experiments the HMM-normalization had considerably lower bias.

The simulated data was obtained by simulating data from normal distributions with variances estimated from a normalized ChIP-chip data; see Materials and Methods. Arguably, real array data are expected to have a considerably higher variability than the simulated data, due to systematic differences between the arrays. Hence, it is reasonable to expect a fairly low signal to noise ratio in real array data. The simulation results suggest that the HMM-normalization outperforms invariant normalization in particular when the signal to noise ratio is low. It follows that the HMM-normalization is expected to have higher sensitivity and lower bias compared to the invariant normalization when applied to real array data.

The above described HMM, invariant and standard normalizations were applied to the three ChIP-chip data sets: E(Z), H3K27me3 and PolII. The normalized data were analysed similarly as the simulated data; see Materials and Methods. Here, the probes were said to be enriched if the log-ratio of their average intensities were above 1.5. The number of probes that were found to be enriched varied depending on which normalization was applied; see Table 1. The HMM-normalization detected more enriched probes than the invariant and standard normalizations. The largest differences were observed in the H3K27me3 data set were the HMM-normalization detected 88% more enriched probes than the invariant normalization and 68% more enriched probes than the standard normalization. The corresponding numbers in the E(Z) and PolII data sets were (9%, 13%) and (10%, 55%) respectively; see Table 1.

**Table 1:** Normalization of three ChIP-chip data sets.

Experiment		StandardNormalization	Invariantnormalization	HMM-assistednormalization
H3K27me3	Enriched (M>1.5)	27004	24110	45317
	M<−1.5	5441	6687	650
E(z)	Enriched (M>1.5)	3927	4057	4440
	M<−1.5	0	0	0
PolII	Enriched (M>1.5)	14435	20392	22395
	M<−1.5	3	0	0

The number of probes identified as being enriched (*i.e.* having an M-value>1.5) and the number of probes with M-values below −1.5 for the H3K27me3, E(z) and PolII experiments. Arguably, the number of false positives in analyses using the HMM and invariant normalizations can be estimated as the number of probes below −1.5.

In order to study the methods' relative sensitivities it is necessary to know the methods' specificities. An indirect observation of experiments' specificity can be obtained by considering the number of probes with log-ratios below the negative cut-off value, which was −1.5 in this case. Assuming that the normalized M-values of the non-enriched probes are symmetrically distributed around zero, one would expect that the number of non-enriched probes below −1.5 would be approximately equal to that above 1.5; if 100 probes fall below −1.5, one would thus expect around 100 false positives. Clearly, the assumption of symmetry is not reasonable when standard normalization techniques are applied to data from skewed experiments, but it should be reasonable for data being normalized with the HMM or invariant normalizations. In the E(z) and PolII experiments, very few probes with log-ratios below −1.5 were detected, suggesting that the vast majority of the probes identified as being enriched are true positives (Table 1). Arguably, this implies that the HMM-normalization as higher sensitivity than the invariant and standard normalizations. Notably, all of the probes identified as being enriched using the standard and invariant approaches were also identified as being enriched when using HMM-assisted normalization. For the H3K27me3 data, the HMM, invariant and standard normalizations had 650, 6687 and 5441 probes with log-ratios below −1.5 respectively; suggesting that the HMM-normalization has considerably higher specificity, and also higher sensitivity, than the invariant normalization. The corresponding specificity cannot be estimated for the standard normalization. One can thus reasonably conclude that the HMM-assisted normalization has considerably higher sensitivity than the standard method, although its specificity may be lower for the H3K27me3 data. It should be noted that the additional normalization step in the HMM-normalization does not cause a linear shift of the M-values, as can be seen in Figure 3D.

We have proposed a hidden Markov model based approach for the identification of unaltered regions (*i.e.* a set of invariant variables). Data from regions classified as unaltered are used to estimate the normalization function. Here, we used the quantile normalization to estimate the normalization function. In the next step all variables are normalized using the estimated normalization function. Our HMM-based method is not platform specific and takes advantage of the dependency structure in datasets where several variables belong to the same *unit* (*e.g.* a chromatin bound region of a protein is represented by several probe enrichment values on the tiling array; a gene will be represented by several read counts in an RNA-seq experiment). Techniques that generate dependent variables include tiling arrays and next generation sequencing based methods. It should be noted that the performance of a HMM is affected by the dependency structure of the data. In particular, experiments with regions containing a large number of altered variables are easily normalized using the HMM-assisted approach. The HMM (or any other invariant method) will generally have difficulties in identifying all altered regions. However, it should be stressed that even if only some of the altered regions are identified (and thus excluded when constructing the normalization function), this will afford better normalization than would be achieved by simply constructing a normalization function based on all of the variables in the data. Applying the HMM-normalization or any other invariant method to an experiment that is not skewed will remove observations that should be included in the estimation of the normalization function. As seen in Figure 2B and Figure S2, the invariant methods can perform considerably worse than the corresponding standard methods when no skewness is present. Therefore, we recommend that the HMM-normalization should be used only if the DSE-test suggests that the experiment is skewed or if there are biological/experimental reasons to assume that the experiment is skewed.

We have shown that the use of standard and previously proposed invariant normalization techniques on skewed experiments has negative effects on downstream interpretations. The asymmetry and the fraction of altered variables have negative and synergistic effects on both bias and sensitivity. To identify such skewed experiments, we have recommended a workflow including the novel DSE-test. The test can easily be used through a web-service. We have also developed a HMM-assisted normalization procedure for use with skewed experiments, which identifies unaltered regions in the data. The entire data set can then be normalized using a normalization function based exclusively on these unaltered regions; we have shown that doing so greatly facilitates the interpretation of simulated and real experimental data.

## Materials and Methods

All data used in this study is MIAME compliant and the raw data is deposited in Gene Expression Omnibus (GEO, www.ncbi.nlm.nih.gov/geo/, accession nrs: GSE29400, GSM454535, GSM454536, GSM409077), a MIAME compliant database.

### Spike-in data

Spike-in data from a two-channel cDNA-microarray experiment with 8 arrays (16 samples) conducted in-house were analysed [12] (GEO: GSE29400). In this work we investigated the performance of skewed one-channel experiments and consequently treated the spike-in data as if it was generated by 16 one-channel arrays. We considered 7760 of the clones on the array, of which 5760 were not regulated and 1920 were up- or down-regulated by a factor of three. Clones with missing values (non-identified spots) were removed from the study. Different data sets were generated from the original data. All sets included all of the non-regulated clones and a set of regulated clones that varied between the sets as described below. All simulated data were analyzed by five normalization methods; a) The standard one-channel quantile normalization [1]. b) The cyclic MA-loess normalization [1]. Both these methods are *standard* normalizations where all genes affect the estimate of the normalization function. c-d) The rank invariant normalization as suggested by Pelz, et al. [9], combined with either one-channel quantile or cyclic MA-loess normalization. e) The modified loess normalization suggested by Risso et al. [5]. In methods a, b and e all clones were used when estimating the normalization function. For the invariant normalizations (c and d) a subset of clones was used to estimate the normalization function. The subset was identified using the method of Pelz et al. [9] with default settings and where the size of the subset was set to 50% of the total number of clones. In addition, ideal normalizations where the normalization function was based only on data from the non-regulated clones were calculated using both MA-loess and quantile normalization algorithms (*ideal MA* and *ideal quantile*). Independent of method the normalization was applied to all 16 samples. The set of regulated clones was selected to mimic different types of skewed experiments. Two parameters were varied: the fraction of regulated clones, and the percentage of up-regulated clones in the set of regulated clones. Four values for the percentage of regulated clones were examined: 5, 10, 15 and 20%. Six values for the percentage of up-regulated clones were examined: 50, 60, 70, 80, 90 and 100%. The normalizations were applied to all experiments defined by the parameter settings above. For each experiment, one hundred sets of regulated clones were randomly selected. The sensitivity of the experiments (at a false positive rate of 0.5%) was estimated as described in [12]; the bias of the regulated clones was estimated as
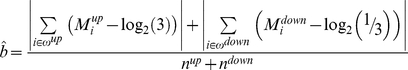
where *M* denotes the log-ratio of a clone's average intensities, ω the set of regulated clones and *n* the number of regulated clones. The indices *up* and *down* refer to up- and down regulated clones. To minimize sampling error, the average bias and sensitivity (taken over the 100 simulated data sets), were used to estimate the experiment's bias and sensitivity. *The relative sensitivity* of a normalization method was calculated as the ratio between the method's sensitivity and the sensitivity achieved using the ideal quantile normalization. *The relative bias* was defined as the increase in bias using the normalization studied compared to the bias observed using the ideal quantile normalization.

### Simulation of data

The simulated data used when evaluating the DSE-test and the HMM-assisted normalization was generated as described below. We aimed to simulate normalized data (data undertaken some form of standard normalization, *e.g.* quantile normalization) from skewed experiments where the positively altered variables are gathered in some regions.

Experiments with *k* treated and reference samples were considered, each with *n* variables. The samples' normalized intensities were simulated as follows: first, the reference intensity 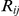 for variable *i* and sample *j* was simulated using a normal distribution with mean 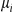 and standard deviation 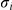, *i.e.* 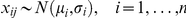, where the parameters 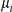 and 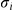 were estimated using data from a ChIP-chip experiment as described below. The corresponding treated intensity 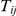, was simulated as
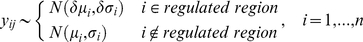
where 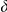 is the ratio of the intensity of the treatment channel to that of the reference. Henceforth, we will refer to 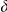 as the effect size. The regions all contained *m* altered variables and were equally spaced so that the distance between any consecutive regions was constant. The number of regions was determined by the percentage of variables that were altered (α) in the experiment.

Data from the two reference samples of the H3K27me3 ChIP-chip experiment [15] were used to estimate the parameters 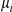 and 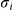, i = 1,…,n. The variables with average intensities above median intensity were selected. For each variable, the mean 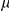 was estimated by the intensity average and 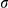 was estimated using the variable's modified standard deviation as described in [20].

Simulated data were generated by altering the percentage of affected variables (*α*), the number of variables (*n*), the number of samples/group (*k*), the effect size (*δ*) and the lengths of the regions (*m*). Individual experiments were defined by specific combinations of parameter settings; each experiment was simulated 1000 times.

### The algorithm used in the dependent DSE-test

Consider an experiment with *n* ***_T_*** treated and *n* ***_R_*** reference samples. The algorithm can be summarized in four steps:

1. All samples are normalized using some standard normalization, *e.g.* the quantile normalization.
2. A heterogeneous set of pairs is created by considering all non-overlapping pairs of treated and reference samples (*i.e.* each sample is only used in one pair). In total, min(*n* ***_T_*** *, n* ***_R_***) non-overlapping heterogeneous pairs can be constructed. For each pair:
  - The log-ratios are calculated.
  - The skewness of the distribution of the log-ratios is estimated by the *qs-coefficient*,
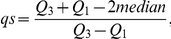
where Q_1_ and Q_3_ are the first and third quartiles of the observed log-ratios.
3. A homogeneous set of pairs is created by considering all non-overlapping pairs of treated samples and reference samples. The number of homogenous non-overlapping pairs that can be constructed from one experiment is floor(*n_T_*/2) + floor(*n_R_*/2). Note, that these pairs can be constructed in several ways. For an experiment with 3 replicates for both treatment (A) and reference (B) it is possible to construct two homogenous non-overlapping pairs, where the pairs can be constructed in nine ways: {A1,A2} and {B1,B2}, {A1,A3} and {B1,B2},…, {A2,A3} and {B2,B3}. Here we only use one of these alternatives.
  - The log-ratios are calculated for each pair.
  - The skewness of the distribution of the log-ratios is estimated using the *qs-coefficient*.
4. Welch's t-test is used to determine whether the mean values of the qs-coefficients are the same for the heterogeneous and homogeneous groups. If there is a significant difference between the groups, then the experiment is said to be skewed.

Here, the Welch t-test can be replaced some other test, *e.g.* some non-parametric alternative. Note that some of the pairs are dependent since the data from each sample is used in both a heterogeneous and a homogeneous set of pairs. It follows that the qs-coefficients are dependent. The independent DSE-test produces independent qs-coefficients, by applying the restriction that a sample is only allowed to be used in one set. The drawback with the independent DSE-test is that it only generates half as many observations as the dependent DSE-test.

### Evaluation of the DSE-tests

Both the independent and the dependent DSE-tests were evaluated similarly. For each experiment, 1000 data sets were simulated. For each data set, a p-value was calculated and the experiment was said to be skewed if its p-value was below 0.05. The experiment's power was estimated by the fraction of sets identified as being skewed. The following parameter settings were considered: *α*: 0, 1, … , 25%, *n*: 10,000, 100,000, *k*: 2 (only the dependent test), 4, 8, 16 and *δ*:1.3, 1.5, 1.8, 2, 4. Note that the length of the regions (*m*) has no influence on the DSE-test.

### Implementation of the DSE-tests

A web application that implements the independent and dependent DSE-tests is available at http://clic-umu.se/Skewness_handling. Users can conduct the asymmetry test on the fly by uploading a tab-delimited text format file containing the log-transformed values for all of their samples. If the data is not normalized, quantile normalization can be applied by the application. The web application will output a p-value for both the dependent and independent DSE-tests and a plot of the distribution of the M-values. More detailed instructions can be obtained from the above web-page. R scripts for performing the DSE-test and the HMM-assisted normalization, together with instructions concerning their use, can also be obtained at this website.

### The algorithm used in the HMM-assisted normalization procedure

Consider an experiment in which the data is skewed in such a way that a large majority of the altered variables are either positively or negatively affected by the treatment and where the fold changes of nearby variables are positively correlated. The HMM-assisted normalization procedure involves four steps:

1. The raw data are normalized using some standard normalization, *e.g.* the quantile normalization.
2. For each variable, the average treated and reference intensities are calculated, together with the *M-value* (*i.e.* the logarithm of the ratio of the average intensities).
3. An HMM with two states is applied to the M-values. Variables belonging to the state whose mean is closest to zero are classified as unaltered variables.
4. The raw data is normalized as in step 1, with the difference that that only variables identified as being unaltered are allowed to influence the normalization; the resulting normalization function is applied to all of the variables.

This HMM method uses a two state model (non-altered, altered) with normally distributed emission probabilities. The HMM estimates were obtained using the *R*-package *HiddenMarkov* [21] with starting parameters ρ, μ_1_, μ_2_, σ_1_ and σ_2_. The value of the parameter ρ (*i.e.* the fraction of altered variables) was guessed. The remaining parameters were estimated under the assumption that the log-ratios were generated by a mixture of two normal distributions with ρ% altered variables, equal variances and mean zero.

### Evaluation of the HMM-assisted normalization procedure

In the downstream analysis, normalized data were used to calculate the M-values. The M-values were smoothed using a moving median of length 21. Variables with smoothed log-ratios above the cut-off were classified as being altered; the cut-off in this case was set to 0.7log_2_(δ). The fraction of true positives and true negatives were used to estimate the experiment's sensitivity and specificity, respectively. The bias was estimated as the difference between the non-smoothed M-value and the logarithm (base 2) of the effect size.

Here 120 experiments were considered (for a detailed description see below) and 10 data sets were simulated for each experiment. For each simulated data set, the sensitivity, specificity and bias were estimated for three normalization methods (HMM, invariant quantile and standard quantile) and the ideal quantile normalization. To minimize simulation error, the average of the estimated measurements (taken over the 10 simulated data sets), were used to estimate the experiments' sensitivity, specificity and bias. The relative sensitivity and the relative bias were calculated as described in; Spike-in data. *The relative specificity* was calculated similarly as the relative sensitivity.

The parameters settings considered in the 120 experiments were: α: 5, 15, 25%, *n*: 100,000, *m*: 50, 100, *k*: 2, 4, 8, 16 and δ: 1.3, 1.5, 2, varied. In addition, an experiment with three different effect sizes (here δ was 1.5, 2 and 4) was considered. Here the effect size within each altered region was constant and the proportion of regions with each effect size was approximately one third.

### Description of the ChIP-chip data

The raw data of E(Z) and H3K27me3 (GEO accession nrs: GSM454535, GSM454536) from Schwartz et al. [15] and the polII data (GEO: GSM409077) from Nègre et al. [16] was mapped to the *Drosophila* reference genome using the Tiling Analysis Software v.1.1 (Affymetrix Inc.). In order to compare the three experiments we removed one replicate of the polII data so that all experiments contained two control and two treatment arrays. The Affymetrix Drosophila Genome Tiling Arrays (Affymetrix Inc.) contains 6.4 million oligonucleotides, of which about half are perfect match probes (PM) and half are mismatch probes (MM). In this study, we only considered the PM probes. On average, there is one PM probe per 35 bp of genomic sequence. All normalizations were performed using the same methods and R-scripts as were used with the simulated data. After normalization, the ChIP vs input ratio data was smoothed using a 700 bp window (on average containing 21 probes), where the value of the centre probe was defined as the median of all probes in the window. Windows containing less than 10 probes were discarded.

## Supporting Information

Figure S1 **Comparison of ideal cyclic MA loess and ideal quantile normalizations.** The sensitivity and bias for the ideal cyclic MA loess normalization compared to the ideal quantile normalization for different percentages of altered clones (% altered) and different distributions of up- and down-regulated clones. (A) The relative sensitivity of the ideal cyclic MA loess normalization compared to the ideal quantile normalization observed at 0.5% false positive rate. (B) The difference in bias between the ideal cyclic MA loess normalization and ideal quantile normalization.
(TIF)

Figure S2 **The effect of violating the primary assumptions.** The sensitivity and bias for the standard cyclic MA-loess normalization and the rank invariant cyclic MA-loess normalization compared to the *ideal quantile normalization* (*i.e.* a quantile normalization where only the non-regulated clones influenced the normalization) for different percentages of altered clones (% altered) and different distributions of up- and down-regulated clones. (A) The relative sensitivity of the standard cyclic MA-loess normalization observed at 0.5% false positive rate (*i.e.* the ratio between the sensitivity observed when the standard cyclic MA-loess and ideal quantile normalization was applied to the data). (B) The relative sensitivity of the invariant cyclic MA-loess normalization at 0.5% false positive rate. (C) The difference in bias between the standard cyclic MA-loess and ideal quantile normalization (D). The difference in bias between the invariant cyclic MA-loess and ideal quantile normalization.
(TIF)

Table S1 **The power of the dependent DSE-test.** The estimated power (at a 5%-significance level) of the dependent DSE-test for different simulated experimental data sets. All estimates were based on 1000 simulated experiments. The power was estimated by the fraction of experiments called skewed. Several experiments were considered. Five different percentages of altered variables (Percent altered) were considered (0, 5, 15, 20 and 25%). Note that the “power” observed when 0% of the variables were altered is an estimate of the false positive rate. The experiments contained data from balanced experiments with *k* biological replicates per treatment; *k* = 2, 4, 8 and 16. Each experiment contained either 10,000 or 100,000 variables, where the altered variables were distributed in regions of length 50 (*i.e. m* = 50). The altered variables were positively affected, with an effect size *δ* = 1.3, 1.5, 1.8, 2 and 4.
(DOCX)

Table S2 **The power of the independent DSE-test.** The estimated power (at a 5%-significance level) of the independent DSE-test for different simulated experimental data sets. All estimates were based on 1000 simulated experiments. The power was estimated by the fraction of experiments called skewed. Several experiments were considered. Five different percentages of altered variables (Percent altered) were considered (0, 5, 15, 20 and 25%). Note that the “power” observed when 0% of the variables were altered is an estimate of the false positive rate. The experiments contained data from balanced experiments with *k* biological replicates per treatment; *k* = 2, 4, 8 and 16. Each experiment contained either 10,000 or 100,000 variables, where the altered variables were distributed in regions of length 50 (*i.e. m* = 50). The altered variables were positively affected, with an effect size *δ* = 1.3, 1.5, 1.8, 2 and 4.
(DOCX)

Table S3 **The relative bias of the HMM, invariant and standard quantile normalizations.** Comparison of the bias of the HMM, invariant and standard quantile normalizations compared to the performance of the ideal quantile normalization. A method's *relative bias* is the difference between its observed bias and the bias observed using the ideal quantile normalization. The methods were evaluated using data simulated from skewed experiments. Three different percentages of altered variables (% altered) were considered (5, 15 and 25%). The experiments contained data from balanced experiments with *k* biological replicates per treatment; *k* = 2, 4, 8 and 16. Each experiment contained 100,000 variables, where the altered variables were distributed in regions of length 50 (*i.e. m* = 50). The altered variables were positively affected, with an effect size *δ* = 1.3, 1.5, 2 and 4. In addition, an experiment with three different effect sizes (denoted *varied*) was considered. Here, approximately one third of the altered variables had an effect size equal to 1.5, 2 and 4 respectively. For each experiment, the estimated relative bias was based on 10 simulated data sets.
(DOCX)

Table S4 **The relative sensitivity of the HMM, invariant and standard quantile normalizations.** Comparison of the sensitivity of the HMM, invariant and standard quantile normalizations relative to the performance of the ideal quantile normalization. A method's *relative sensitivity* is the ratio between its observed sensitivity and the sensitivity observed using the ideal normalization. The methods were evaluated using data simulated from skewed experiments. Three different percentages of altered variables (% altered) were considered (5, 15 and 25%). The experiments contained data from balanced experiments with *k* biological replicates per treatment; *k* = 2, 4, 8 and 16. Each experiment contained 100,000 variables, where the altered variables were distributed in regions of length 50 (*i.e. m* = 50). The altered variables were positively affected, with an effect size *δ* = 1.3, 1.5, 2 and 4. In addition, an experiment with three different effect sizes (denoted *varied*) was considered. Here, approximately one third of the altered variables had an effect size equal to 1.5, 2 and 4 respectively. A variable with an average M-value above the cut-off (*i.e.* 0.7log_2_(*δ*)) was called altered. For each experiment, the estimated relative sensitivity was based on 10 simulated data sets.
(DOCX)

Table S5 **The relative specificity of the HMM, invariant and standard quantile normalizations.** Comparison of the specificity of the HMM, invariant and standard quantile normalizations relative to the performance of the ideal quantile normalization. A method's *relative specificity* is the ratio between its observed specificity and the specificity observed using the ideal normalization. The methods were evaluated using data simulated from skewed experiments. Three different percentages of altered variables (% altered) were considered (5, 15 and 25%). The experiments contained data from balanced experiments with *k* biological replicates per treatment; *k* = 2, 4, 8 and 16. Each experiment contained 100,000 variables, where the altered variables were distributed in regions of length 50 (*i.e. m* = 50). The altered variables were positively affected, with an effect size *δ* = 1.3, 1.5, 2 and 4. In addition, an experiment with three different effect sizes (denoted *varied*) was considered. Here, approximately one third of the altered variables had an effect size equal to 1.5, 2 and 4 respectively. A variable with an average M-value above the cut-off (*i.e.* 0.7log_2_(*δ*)) was called altered. For each experiment, the estimated relative specificity was based on 10 simulated data sets.
(DOCX)
