# Supplementary figures and images for: Normalization of High Dimensional Genomics Data Where the Distribution of the Altered Variables Is Skewed
Source: PLoS One. 2011 Nov 22;6(11):e27942. doi: 10.1371/journal.pone.0027942 (PMC3222656; doi:10.1371/journal.pone.0027942)

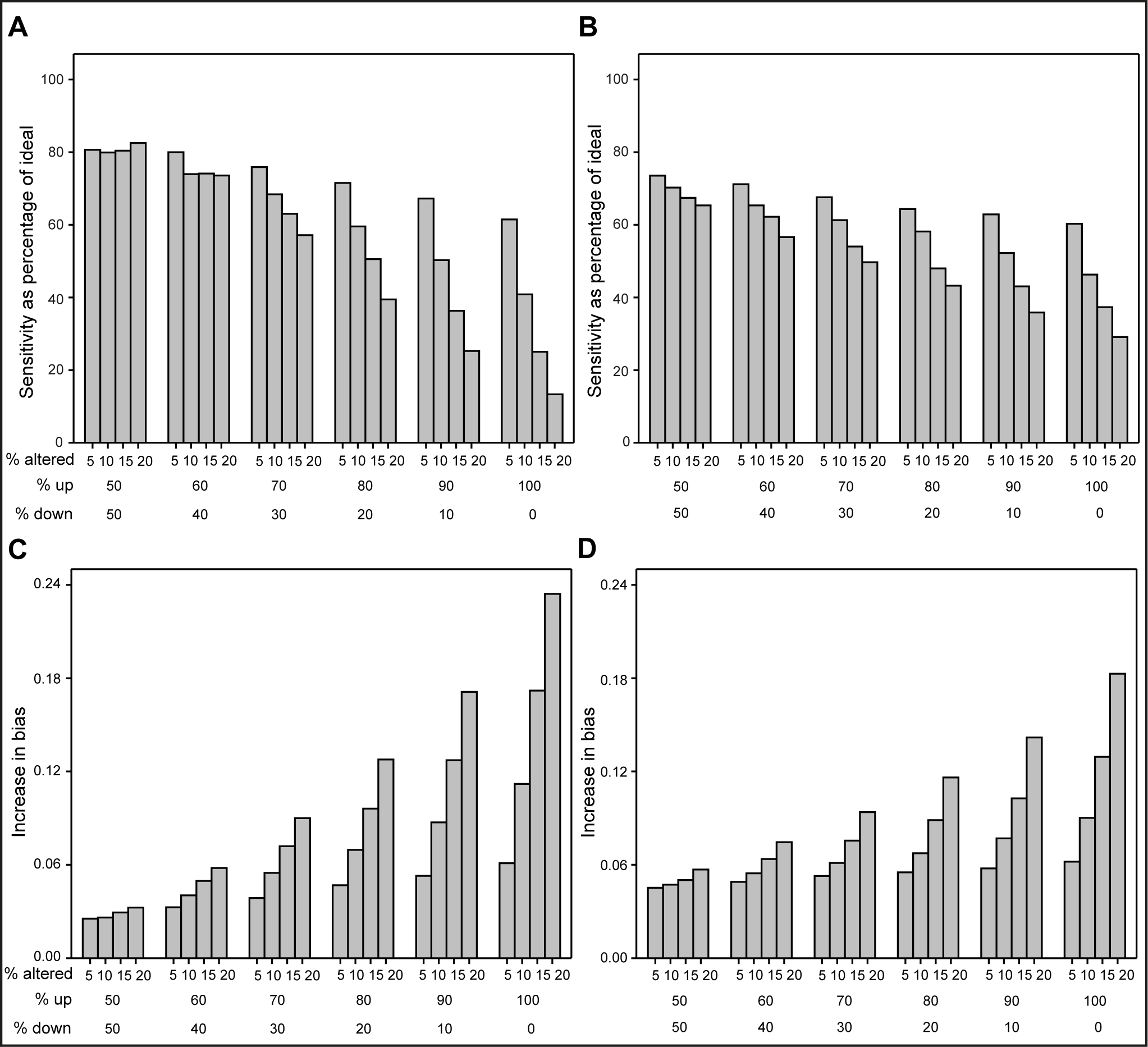

Supplement: Figure S1 — Comparison of ideal cyclic MA loess and ideal quantile normalizations. The sensitivity and bias for the ideal cyclic MA loess normalization compared to the ideal quantile normalization for different percentages of altered clones (% altered) and different distributions of up- and down-regulated clones. (A) The relative sensitivity of the ideal cyclic MA loess normalization compared to the ideal quantile normalization observed at 0.5% false positive rate. (B) The difference in bias between the ideal cyclic MA loess normalization and ideal quantile normalization. (TIF) [file pone.0027942.s001.tif]

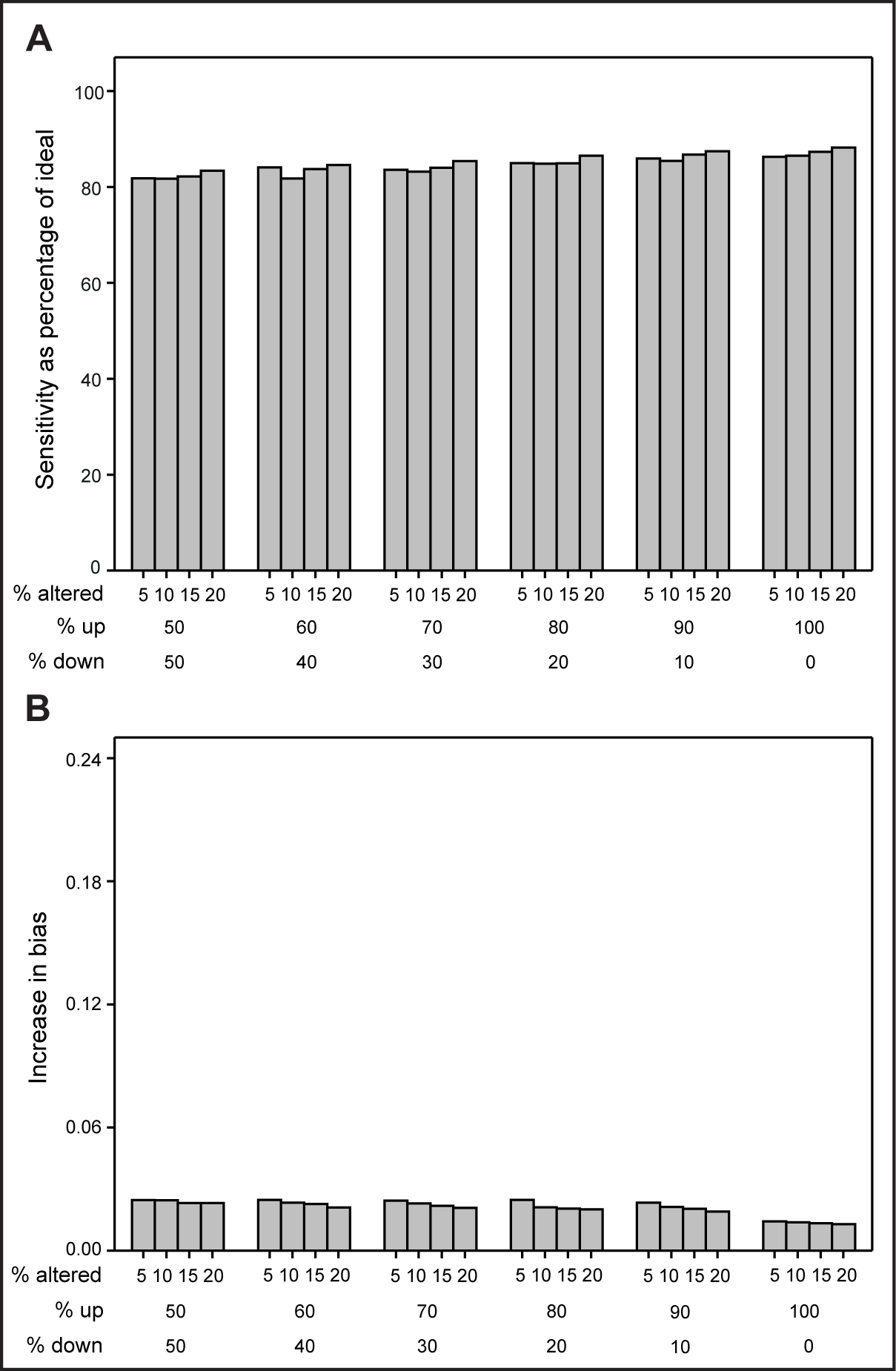

Supplement: Figure S2 — The effect of violating the primary assumptions. The sensitivity and bias for the standard cyclic MA-loess normalization and the rank invariant cyclic MA-loess normalization compared to the ideal quantile normalization (i.e. a quantile normalization where only the non-regulated clones influenced the normalization) for different percentages of altered clones (% altered) and different distributions of up- and down-regulated clones. (A) The relative sensitivity of the standard cyclic MA-loess normalization observed at 0.5% false positive rate (i.e. the ratio between the sensitivity observed when the standard cyclic MA-loess and ideal quantile normalization was applied to the data). (B) The relative sensitivity of the invariant cyclic MA-loess normalization at 0.5% false positive rate. (C) The difference in bias between the standard cyclic MA-loess and ideal quantile normalization (D). The difference in bias between the invariant cyclic MA-loess and ideal quantile normalization. (TIF) [file pone.0027942.s002.tif]
