# Supplementary material for: Normalization of High Dimensional Genomics Data Where the Distribution of the Altered Variables Is Skewed
Source: PLoS One. 2011 Nov 22;6(11):e27942. doi: 10.1371/journal.pone.0027942 (PMC3222656; doi:10.1371/journal.pone.0027942)
Supplement: Table S2 — The power of the independent DSE-test. The estimated power (at a 5%-significance level) of the independent DSE-test for different simulated experimental data sets. All estimates were based on 1000 simulated experiments. The power was estimated by the fraction of experiments called skewed. Several experiments were considered. Five different percentages of altered variables (Percent altered) were considered (0, 5, 15, 20 and 25%). Note that the “power” observed when 0% of the variables were altered is an estimate of the false positive rate. The experiments contained data from balanced experiments with k biological replicates per treatment; k = 2, 4, 8 and 16. Each experiment contained either 10,000 or 100,000 variables, where the altered variables were distributed in regions of length 50 (i.e. m = 50). The altered variables were positively affected, with an effect size δ = 1.3, 1.5, 1.8, 2 and 4. (DOCX) [file pone.0027942.s004.docx]

Table S2

| Number of variables | Experimental design  (treatment-control) | Effect size (δ) | Percent altered | | | | | |
| --- | --- | --- | --- | --- | --- | --- | --- | --- |
|  |  |  | 0% | 5% | 10% | 15% | 20% | 25% |
| 10,000 | 2-2 | 1.3 | n/a | n/a | n/a | n/a | n/a | n/a |
|  |  | 1.5 | n/a | n/a | n/a | n/a | n/a | n/a |
|  |  | 1.8 | n/a | n/a | n/a | n/a | n/a | n/a |
|  |  | 2 | n/a | n/a | n/a | n/a | n/a | n/a |
|  |  | 4 | n/a | n/a | n/a | n/a | n/a | n/a |
|  | 4-4 | 1.3 | 2.8 | 2 | 4 | 4 | 4 | 5 |
|  |  | 1.5 | 2.5 | 4 | 13 | 18 | 25 | 29 |
|  |  | 1.8 | 2.9 | 10 | 28 | 51 | 65 | 77 |
|  |  | 2 | 1.9 | 11 | 35 | 66 | 85 | 92 |
|  |  | 4 | 2.7 | 14 | 50 | 80 | 96 | 100 |
|  | 8-8 | 1.3 | 3.2 | 6 | 14 | 18 | 24 | 26 |
|  |  | 1.5 | 3.5 | 24 | 61 | 89 | 99 | 99 |
|  |  | 1.8 | 3.8 | 55 | 99 | 100 | 100 | 100 |
|  |  | 2 | 4.0 | 64 | 100 | 100 | 100 | 100 |
|  |  | 4 | 3.9 | 79 | 100 | 100 | 100 | 100 |
|  | 16-16 | 1.3 | 4.9 | 13 | 27 | 44 | 54 | 56 |
|  |  | 1.5 | 4.3 | 48 | 96 | 100 | 100 | 100 |
|  |  | 1.8 | 4.3 | 92 | 100 | 100 | 100 | 100 |
|  |  | 2 | 5.0 | 98 | 100 | 100 | 100 | 100 |
|  |  | 4 | 4.2 | 100 | 100 | 100 | 100 | 100 |
| 100,000 | 2-2 | 1.3 | n/a | n/a | n/a | n/a | n/a | n/a |
|  |  | 1.5 | n/a | n/a | n/a | n/a | n/a | n/a |
|  |  | 1.8 | n/a | n/a | n/a | n/a | n/a | n/a |
|  |  | 2 | n/a | n/a | n/a | n/a | n/a | n/a |
|  |  | 4 | n/a | n/a | n/a | n/a | n/a | n/a |
|  | 4-4 | 1.3 | 2.6 | 4 | 14 | 21 | 25 | 29 |
|  |  | 1.5 | 1.8 | 24 | 57 | 74 | 81 | 86 |
|  |  | 1.8 | 2.8 | 47 | 87 | 98 | 100 | 100 |
|  |  | 2 | 2.5 | 59 | 93 | 99 | 100 | 100 |
|  |  | 4 | 2.3 | 66 | 96 | 100 | 100 | 100 |
|  | 8-8 | 1.3 | 4.8 | 30 | 75 | 94 | 98 | 98 |
|  |  | 1.5 | 3.6 | 96 | 100 | 100 | 100 | 100 |
|  |  | 1.8 | 4.4 | 100 | 100 | 100 | 100 | 100 |
|  |  | 2 | 4.0 | 100 | 100 | 100 | 100 | 100 |
|  |  | 4 | 3.1 | 100 | 100 | 100 | 100 | 100 |
|  | 16-16 | 1.3 | 4.6 | 68 | 99 | 100 | 100 | 100 |
|  |  | 1.5 | 5.6 | 100 | 100 | 100 | 100 | 100 |
|  |  | 1.8 | 5.5 | 100 | 100 | 100 | 100 | 100 |
|  |  | 2 | 5.0 | 100 | 100 | 100 | 100 | 100 |
|  |  | 4 | 3.8 | 100 | 100 | 100 | 100 | 100 |
