# Supplementary material for: Normalization of High Dimensional Genomics Data Where the Distribution of the Altered Variables Is Skewed
Source: PLoS One. 2011 Nov 22;6(11):e27942. doi: 10.1371/journal.pone.0027942 (PMC3222656; doi:10.1371/journal.pone.0027942)
Supplement: Table S3 — The relative bias of the HMM, invariant and standard quantile normalizations. Comparison of the bias of the HMM, invariant and standard quantile normalizations compared to the performance of the ideal quantile normalization. A method's relative bias is the difference between its observed bias and the bias observed using the ideal quantile normalization. The methods were evaluated using data simulated from skewed experiments. Three different percentages of altered variables (% altered) were considered (5, 15 and 25%). The experiments contained data from balanced experiments with k biological replicates per treatment; k = 2, 4, 8 and 16. Each experiment contained 100,000 variables, where the altered variables were distributed in regions of length 50 (i.e. m = 50). The altered variables were positively affected, with an effect size δ = 1.3, 1.5, 2 and 4. In addition, an experiment with three different effect sizes (denoted varied) was considered. Here, approximately one third of the altered variables had an effect size equal to 1.5, 2 and 4 respectively. For each experiment, the estimated relative bias was based on 10 simulated data sets. (DOCX) [file pone.0027942.s005.docx]

Table S3

| Experimental design  (treatment-reference) | Effect size (*δ*) | Percent altered | | | | | | | | |
| --- | --- | --- | --- | --- | --- | --- | --- | --- | --- | --- |
|  |  | 5% | | | 15% | | | 25% | | |
|  |  | S | I | HMM | S | I | HMM | S | I | HMM |
| 2-2 | 1.3 | 0,019 | 0,012 | 0,009 | 0,059 | 0,042 | 0,002 | 0,097 | 0,077 | 0,002 |
| 2-2 | 1.5 | 0,031 | 0,013 | 0,002 | 0,093 | 0,047 | 0,002 | 0,154 | 0,093 | 0,002 |
| 2-2 | 2 | 0,059 | 0,013 | 0,002 | 0,172 | 0,046 | 0,002 | 0,280 | 0,094 | 0,002 |
| 2-2 | 4 | 0,155 | 0,027 | 0,002 | 0,430 | 0,095 | 0,001 | 0,660 | 0,173 | 0,001 |
| 2-2 | Varied | 0,072 | 0,014 | 0,000 | 0,217 | 0,053 | 0,003 | 0,349 | 0,102 | 0,023 |
| 4-4 | 1.3 | 0,020 | 0,011 | 0,001 | 0,058 | 0,036 | 0,001 | 0,097 | 0,069 | 0,001 |
| 4-4 | 1.5 | 0,031 | 0,010 | 0,001 | 0,093 | 0,036 | 0,001 | 0,154 | 0,074 | 0,001 |
| 4-4 | 2 | 0,059 | 0,012 | 0,001 | 0,172 | 0,040 | 0,001 | 0,280 | 0,078 | 0,001 |
| 4-4 | 4 | 0,156 | 0,032 | 0,001 | 0,429 | 0,102 | 0,000 | 0,660 | 0,180 | 0,000 |
| 4-4 | Varied | 0,072 | 0,014 | 0,001 | 0,218 | 0,050 | 0,000 | 0,349 | 0,095 | 0,003 |
| 8-8 | 1.3 | 0,020 | 0,009 | 0,001 | 0,058 | 0,031 | 0,001 | 0,097 | 0,060 | 0,001 |
| 8-8 | 1.5 | 0,031 | 0,009 | 0,001 | 0,093 | 0,029 | 0,001 | 0,154 | 0,059 | 0,001 |
| 8-8 | 2 | 0,059 | 0,011 | 0,001 | 0,172 | 0,039 | 0,001 | 0,280 | 0,072 | 0,001 |
| 8-8 | 4 | 0,155 | 0,031 | 0,000 | 0,430 | 0,106 | 0,001 | 0,662 | 0,181 | 0,000 |
| 8-8 | Varied | 0,072 | 0,013 | 0,000 | 0,218 | 0,050 | 0,000 | 0,349 | 0,089 | 0,001 |
| 16-16 | 1.3 | 0,020 | 0,007 | 0,000 | 0,059 | 0,026 | 0,001 | 0,097 | 0,052 | 0,001 |
| 16-16 | 1.5 | 0,031 | 0,008 | 0,000 | 0,093 | 0,025 | 0,000 | 0,154 | 0,050 | 0,001 |
| 16-16 | 2 | 0,059 | 0,012 | 0,000 | 0,172 | 0,038 | 0,000 | 0,280 | 0,069 | 0,000 |
| 16-16 | 4 | 0,156 | 0,034 | 0,000 | 0,429 | 0,105 | 0,001 | 0,661 | 0,180 | 0,000 |
| 16-16 | Varied | 0,072 | 0,013 | 0,000 | 0,218 | 0,048 | 0,000 | 0,349 | 0,085 | 0,000 |
