# Supplementary material for: Normalization of High Dimensional Genomics Data Where the Distribution of the Altered Variables Is Skewed
Source: PLoS One. 2011 Nov 22;6(11):e27942. doi: 10.1371/journal.pone.0027942 (PMC3222656; doi:10.1371/journal.pone.0027942)
Supplement: Table S5 — The relative specificity of the HMM, invariant and standard quantile normalizations. Comparison of the specificity of the HMM, invariant and standard quantile normalizations relative to the performance of the ideal quantile normalization. A method's relative specificity is the ratio between its observed specificity and the specificity observed using the ideal normalization. The methods were evaluated using data simulated from skewed experiments. Three different percentages of altered variables (% altered) were considered (5, 15 and 25%). The experiments contained data from balanced experiments with k biological replicates per treatment; k = 2, 4, 8 and 16. Each experiment contained 100,000 variables, where the altered variables were distributed in regions of length 50 (i.e. m = 50). The altered variables were positively affected, with an effect size δ = 1.3, 1.5, 2 and 4. In addition, an experiment with three different effect sizes (denoted varied) was considered. Here, approximately one third of the altered variables had an effect size equal to 1.5, 2 and 4 respectively. A variable with an average M-value above the cut-off (i.e. 0.7log2(δ)) was called altered. For each experiment, the estimated relative specificity was based on 10 simulated data sets. (DOCX) [file pone.0027942.s007.docx]

Table S5

| Experimental design  (treatment-reference) | Effect size (*δ*) | Percent altered | | | | | | | | |
| --- | --- | --- | --- | --- | --- | --- | --- | --- | --- | --- |
|  |  | 5% | | | 15% | | | 25% | | |
|  |  | S | I | HMM | S | I | HMM | S | I | HMM |
| 2-2 | 1.3 | 100,0 | 100,0 | 100,0 | 99,7 | 99,9 | 100,0 | 98,8 | 99,6 | 100,0 |
| 2-2 | 1.5 | 100,0 | 100,0 | 100,0 | 100,0 | 100,0 | 100,0 | 100,0 | 100,1 | 100,0 |
| 2-2 | 2 | 100,0 | 100,0 | 100,0 | 100,0 | 100,0 | 100,0 | 100,0 | 100,0 | 100,0 |
| 2-2 | 4 | 100,0 | 100,0 | 100,0 | 100,0 | 100,0 | 100,0 | 100,0 | 100,0 | 100,0 |
| 2-2 | Varied | 100,0 | 100,0 | 100,0 | 99,7 | 100,0 | 100,0 | 90,3 | 100,1 | 100,1 |
| 4-4 | 1.3 | 100,0 | 100,0 | 100,0 | 100,0 | 100,0 | 100,0 | 100,0 | 100,1 | 100,0 |
| 4-4 | 1.5 | 100,0 | 100,0 | 100,0 | 100,0 | 100,0 | 100,0 | 100,0 | 100,0 | 100,0 |
| 4-4 | 2 | 100,0 | 100,0 | 100,0 | 100,0 | 100,0 | 100,0 | 100,0 | 100,0 | 100,0 |
| 4-4 | 4 | 100,0 | 100,0 | 100,0 | 100,0 | 100,0 | 100,0 | 100,0 | 100,0 | 100,0 |
| 4-4 | Varied | 100,0 | 100,0 | 100,0 | 100,0 | 100,0 | 100,0 | 95,1 | 100,0 | 100,0 |
| 8-8 | 1.3 | 100,0 | 100,0 | 100,0 | 100,0 | 100,0 | 100,0 | 100,1 | 100,1 | 100,0 |
| 8-8 | 1.5 | 100,0 | 100,0 | 100,0 | 100,0 | 100,0 | 100,0 | 100,0 | 100,0 | 100,0 |
| 8-8 | 2 | 100,0 | 100,0 | 100,0 | 100,0 | 100,0 | 100,0 | 100,0 | 100,0 | 100,0 |
| 8-8 | 4 | 100,0 | 100,0 | 100,0 | 100,0 | 100,0 | 100,0 | 100,0 | 100,0 | 100,0 |
| 8-8 | Varied | 100,0 | 100,0 | 100,0 | 100,0 | 100,0 | 100,0 | 97,7 | 100,0 | 100,0 |
| 16-16 | 1.3 | 100,0 | 100,0 | 100,0 | 100,0 | 100,0 | 100,0 | 100,0 | 100,0 | 100,0 |
| 16-16 | 1.5 | 100,0 | 100,0 | 100,0 | 100,0 | 100,0 | 100,0 | 100,0 | 100,0 | 100,0 |
| 16-16 | 2 | 100,0 | 100,0 | 100,0 | 100,0 | 100,0 | 100,0 | 100,0 | 100,0 | 100,0 |
| 16-16 | 4 | 100,0 | 100,0 | 100,0 | 100,0 | 100,0 | 100,0 | 100,0 | 100,0 | 100,0 |
| 16-16 | Varied | 100,0 | 100,0 | 100,0 | 100,0 | 100,0 | 100,0 | 98,5 | 100,0 | 100,0 |
